# Analysis of Sirtuin 1 and Sirtuin 3 at Enzyme and Protein Levels in Human Breast Milk during the Neonatal Period

**DOI:** 10.3390/metabo11060348

**Published:** 2021-05-29

**Authors:** Kristina Hase, Laura Stahmer, Hadeel Shammas, Corinna Peter, Bettina Bohnhorst, Anibh Martin Das

**Affiliations:** 1 Clinic for Pediatric Kidney-, Liver- and Metabolic Diseases, Hannover Medical School, 30625 Hannover, Germany; kristina.hase@stud.mh-hannover.de (K.H.); laura.k.c.stahmer@stud.mh-hannover.de (L.S.); shammas.hadeel@mh-hannover.de (H.S.); 2 Clinic for Pediatric Pneumology, Allergology and Neonatology, Hannover Medical School, 30625 Hannover, Germany; peter.corinna@mh-hannover.de (C.P.); bohnhorst.bettina@mh-hannover.de (B.B.)

**Keywords:** breast milk, fetal programming, human, lactation, neonates, sirtuins

## Abstract

Breast feeding is regarded as the preferred nutrition modality for children during the first few months of life. It not only furthers growth and development but also is supposed to impact later life. The first 1000 days are regarded as a critical window for development, even beyond infancy. The physiological basis underlying this beneficial effect is not clear. Sirtuins are important regulatory proteins of metabolism and are supposed to play a critical role in ageing and longevity as well as in diseases. In the present study, we developed novel methods to assay sirtuin 1 and sirtuin 3 at enzyme activity (via fluorometry) and protein levels (by Western blot) in the aqueous phase and in the cell pellet of human breast milk and assessed the impact of ongoing lactation during the neonatal period. Sirtuin activities in the aqueous phase were negatively correlated with the duration of lactation in the neonatal period. There was no correlation of sirtuin activities in the cell pellet with the duration of lactation. The amounts of sirtuin 1 and sirtuin 3 measured by Western blot were negatively correlated with the lactation period.

## 1. Introduction

Breast feeding is regarded as the optimal nutrition modality for neonates and infants in terms of physiological growth and development. The World Health Organization (WHO) recommends breast milk as the main nutrition for at least 6 months after birth [1]. Breast milk protects babies from infection and promotes immunological competence [2,3]. Furthermore, neonatal and infant nutrition is supposed to have an impact on later life with respect to health and disease. In his seminal work, D. Barker coined the term ‘fetal programming’, primarily referring to the fetal origins of coronary heart disease [4]. Later concepts regard the first 1000 days (i.e., the period from conception to the second birthday) as a critical window for growth, development, health, and well-being, even beyond this period [5,6]. The physiological basis underlying this early programming is not completely understood. 

There are multiple studies about the influence of perinatal, maternal, and environmental factors on breast milk composition; especially, the role of human milk oligosaccharides has been addressed [7,8,9,10]. Only little is known about sirtuins in breast milk.

Sirtuins are class III histone deacetylases and consist of seven subtypes (SIRT 1–7) with different subcellular localizations and functions. Their dependency on NAD+ directly links them to cellular energy metabolism where they function as regulatory proteins. They are known to modulate metabolism, cell signaling, ageing, and longevity [11,12,13,14,15,16,17]. In particular, sirtuin 1 is known to be involved in the regulation of lipid and glucose metabolism, while sirtuin 3 regulates mitochondrial energy metabolism [18,19]. Moreover, several studies have linked sirtuins to neurodegenerative diseases, and metabolic changes such as caloric restriction trigger alterations of sirtuin 2 and sirtuin 3 [15,17]. Furthermore, both sirtuin 1 and sirtuin 3 are known to play an important role in oxidative stress and redox signaling [20,21]. During pregnancy and birth, as well as after birth, oxidative stress by reactive oxygen species (ROS) may occur [22,23]. 

Recently, we have shown that nutrition may impact sirtuins 1 and 3 in human adults [24]. Thus, sirtuins in breast milk are strong candidates for mediating the positive impact of breast feeding on the health of neonates and children. A recent review suggested a role of sirtuins in pre- and postnatal programming, which may result in (metabolic) diseases during later life [25]. Additionally, sirtuins 1, 2, and 3 have been shown to modulate neurodegeneration in the enteric nervous system [26] and may impact the brain by the microbiota–gut–brain axis, which has been suggested to be involved in priming important organ systems via early-life nutrition [27]. Sirtuin 1 was reported to regulate the gut microbiota [28], sirtuin 2 was found to maintain gut homeostasis [29], while sirtuin 1 is supposed to be involved in the pathophysiology of necrotizing enterocolitis [30].

To the best of our knowledge, little is known about sirtuins in human breast milk. A recent study described the impact of perinatal factors on the amount of sirtuin 3 in early human breast milk; however, the enzymatic activity of sirtuin 3 as a functional parameter was not reported [22]. 

Animal experimentation showed that the administration of resveratrol, an activator of sirtuins, to mothers and neonatal offspring was able to prevent neurological dysfunction and metabolic syndrome induced by a high-fat diet in mothers and neonates [31,32]. 

The constituents of breast milk are known to change with postnatal age [33,34]. These observations prompted us to develop analytical methods to characterize the amount and function of the most relevant sirtuins, namely, sirtuin 1 and sirtuin 3, in human breast milk, both in the aqueous phase and in the pellet that contains the cellular constituents of breast milk. We also addressed changes in sirtuin 1 and sirtuin 3 during the first 4 weeks of lactation, i.e., in the neonatal period.

We show that sirtuin 1 and sirtuin 3 can be measured in human breast milk at enzyme activity and protein levels in the cellular pellet. While the enzyme activities could be measured in the aqueous phase, protein measurements were not possible in the aqueous phase due to unspecific interference. We found a negative correlation between sirtuin 1 and sirtuin 3 activities in the aqueous phase of breast milk and the duration of lactation during the neonatal period. Enzyme activities in the cell pellet were not correlated with the duration of lactation. 

## 2. Results

Milk samples were collected from 10 mothers on postnatal days 7, 14, 21, and 28 ± 1, respectively. Details of pregnancies are summarized in Table Eight of these mothers delivered preterm (M1, M4, M5, M6, M7, M8, M9, and M10, see Table 1a,b), and two of them gave birth at term (M2 and M3, see Table 1a,b). The mean age of the preterm mothers was 33.8 ± 4.4 years, and that of the mothers delivering at term was 32.5 ± 6.4 years. The mothers were considered healthy, as none of them suffered from metabolic diseases (diabetes mellitus) or metabolic pregnancy complications, e.g., preeclampsia, HELLP syndrome, as detailed in Table 1a,b.

### 2.1. Sirtuin 1 Enzymatic Activity

Technical repeats of sirtuin 1-activity varied by about 5%. 

The mean activity of sirtuin 1 in the pellet at different days was about 0.06 U/µL, while it varied in the supernatant between 0.02 and 0.04 U/µL. 

**Figure 1:**
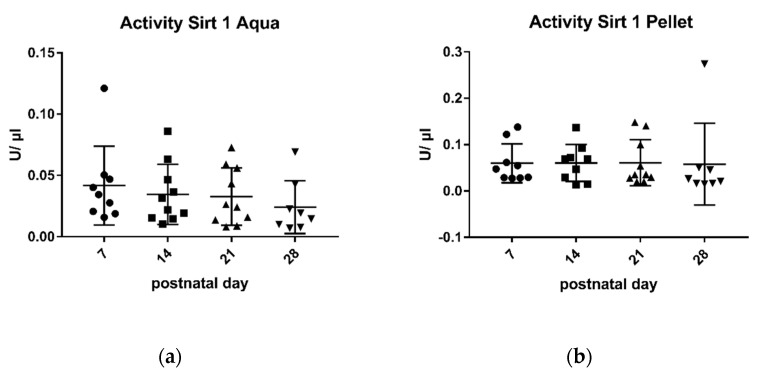
Enzymatic activity of sirtuin 1 in aqueous phase (**a**) and pellet (**b**). Mean ± SD. Day 7 n = 9, day 14 aqua n = 10 and pellet n = 9, day 21 n = 10, day 28 n = There was a negative correlation between sirtuin 1-activity in the aqueous phase and the duration of lactation (r: −0.97). Different scatter points present different postnatal age.

**Figure 2:**
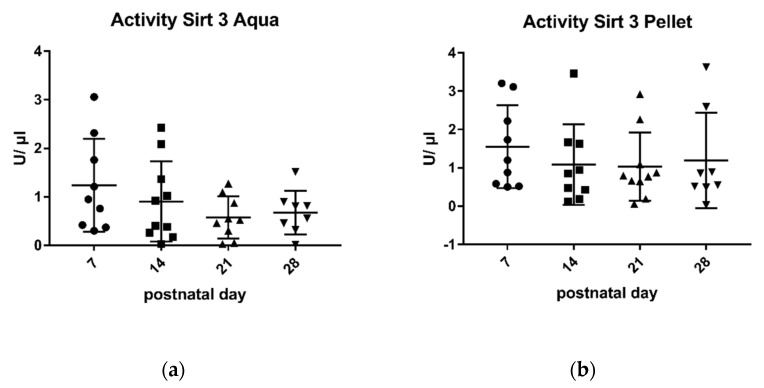
Enzymatic activity of sirtuin 3 in aqueous phase (**a**) and in pellet (**b**). Mean ± SD. Day 7 n = 9, day 14 aqua n = 10 and pellet n = 9, day 21 pellet n = 10 and aqua n = 9, day 28 n = There was a negative correlation between sirtuin 3-activity in the aqueous phase and the duration of lactation (r: −0.88).

Sirtuin 1-activity in breast milk was much lower than sirtuin 3-activity, both in the pellet and in the aqueous phase (Figure 1 and Figure 2). 

Sirtuin 1-activity was the highest 7 days after birth and decreased, with a longer lactation period, in the aqueous phase. 

There was a good correlation between sirtuin 1-activity in the aqueous phase and duration of lactation (r: −0.97, * *p*: 0.029) (Figure 1). 

Sirtuin 1-activity in the pellet did not parallel enzyme activity in the aqueous phase, there was no correlation of enzyme activity in the pellet and duration of lactation, and no significant differences during the lactation period were observed (r: −0.4) (Figure 1). 

### 2.2. Sirtuin 3 Enzymatic Activity

The variation of technical repeats was typically about 5%**.**

The mean activity of sirtuin 3 in the pellet varied between 1 U/µL and 1.5 U/µL at different days of lactation, and in the supernatant between 0.5 U/µL and 1.2 U/µL (Figure 2). Similar to sirtuin 1, the enzymatic activity of sirtuin 3 was the highest at day 7 and subsequently showed a negative correlation with the duration of lactation (r: −0.88) (Figure 2). Regarding sirtuin 3 activity in the pellet, no significant differences were found on different days of lactation (r: −0.55) (Figure 2).

### 2.3. Sirtuin 1 Western Blot

**Figure 3:**
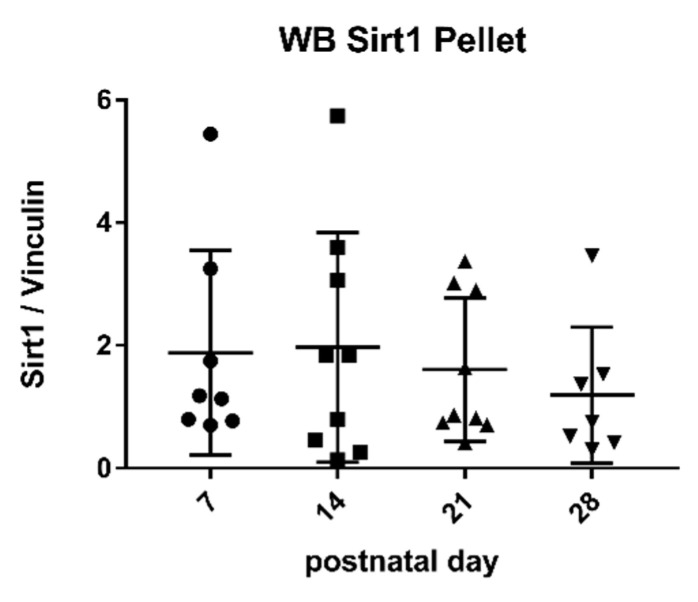
Protein levels of sirtuin 1 in the pellet. Mean ± SD. Day 7 n = 8, day 14 n = 9, day a21 n = 9, day 28 n = The SIRT/Vinculin ratio correlated negatively with the duration of lactation (r: −0.89).

**Figure 4:**
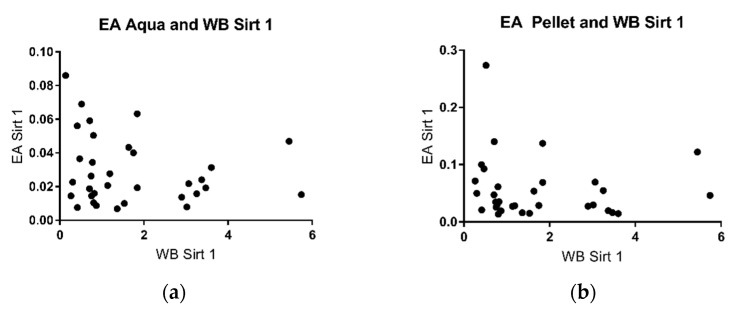
SIRT 1/Vinculin ratio in the pellet in correlation with the enzymatic activity in the aqueous phase (r: −0.165) (**a**) and in the cell pellet (r: −0.24) (**b**).

Western Blot analysis indicated that expressed protein levels of SIRT 1 were detectable in the pellet by using specific antibodies against the enzyme (Figure 3). The SIRT/Vinculin ratio in the pellet did not correlate with enzymatic activity in the aqueous phase as well as in the pellet (Figure 4) but with the duration of lactation (r: −0.89) (Figure 3).

### 2.4. Sirtuin 3 Western Blot

**Figure 5:**
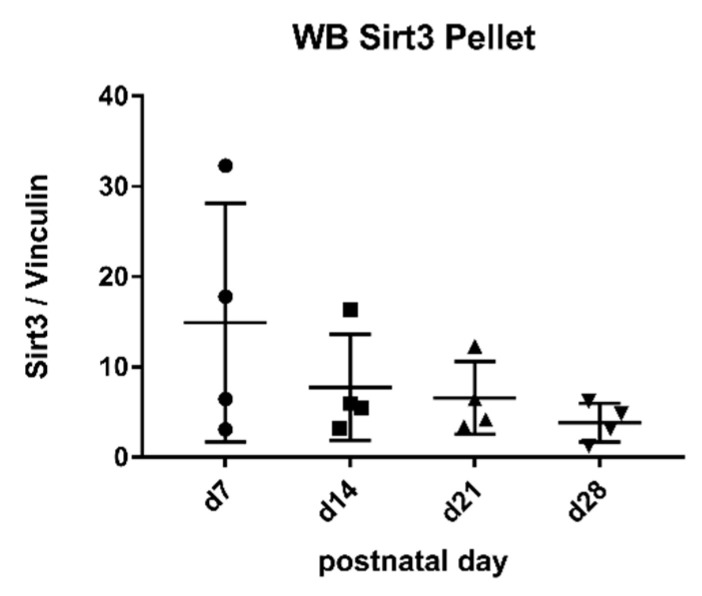
Protein levels of sirtuin 3 in the pellet. Mean ± SD. All days, n = The SIRT/Vinculin ratio showed good correlation with the duration of lactation (r: −0.94).

**Figure 6:**
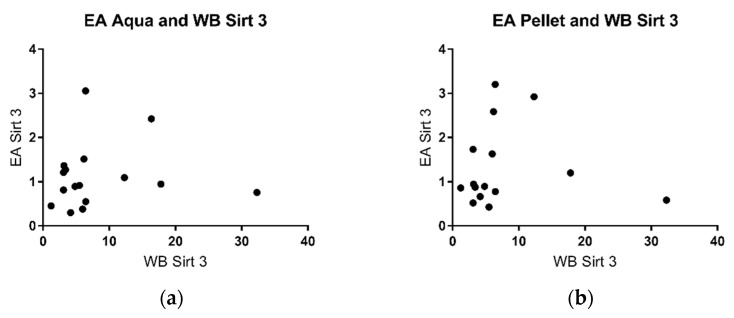
SIRT 3/Vinculin ratio in correlation with the enzymatic activity in the aqueous phase (**a**) (r: 0.162) and in the cell pellet (**b**) (r: 0.2).

SIRT 3 could be detected in the cell pellet using specific antibodies (Figure 5). There was good correlation with the duration of lactation (r: −0.94) (Figure 5). There was poor correlation of sirtuin 3 activity in the aqueous phase as well as the cell pellet with sirtuin protein levels in the pellet (Figure 6).

## 3. Discussion

Breast milk is regarded as the preferred nutrition for children in the first few months of life, with beneficial effects beyond infancy [5,6,35]. Sirtuins especially protect mitochondrial physiology, which is important not only for energy homeostasis but also in the context of ageing and organ dysfunction [36]. Preterms are protected from necrotizing enterocolitis as well as infections and retinopathy if they are fed breast milk [37]. Sirtuin 1- related pathways have been implicated in the protective effect of the probiotic *Saccharomyces boulardii* regarding necrotizing enterocolitis [30].

Human breast milk contains a plethora of constituents, which may mediate beneficial effects. The constituents of breast milk may both protect and nourish the offspring, and many components may even have dual roles [35]. The most abundantly studied function of breast milk is immunological protection of the child [38,39,40]. Lactose, fat, and oligosaccharides are the most abundant constituents of human breast milk [8]. Breast milk contains macronutrients, roughly 7% of carbohydrates, 5% of lipids, 0.9% of protein, and 0.2% of minerals emulsified in the aqueous phase [41,42]. Furthermore, miRNAs are constituents of breast milk that may have regulatory functions by serving as post-transcriptional regulators in the gut or when systemically taken up by the child and/or may serve as nutrients [43]. Human milk contains cells of both maternal and bacterial origin. These cells are important regulatory constituents of breast milk and may have an impact on the aqueous phase of the milk [38,44]. Therefore, both the cellular and the aqueous phase may be important for mediating the beneficial effect of breast feeding, which prompted us to study not only the aqueous phase but also the cellular phase of human milk. So far, the metabolic functions of breast milk cells are not well characterized [45].

Sirtuins have numerous functions in the organism, ranging from the regulation of metabolism to tumorigenesis, cell signaling, ageing, and longevity [11,12,13,14,15,16,17]. This recently prompted us to study the impact of nutrition and exercise on sirtuins in human adult blood [24]. The aim of the present study was to develop analytical methods for the determination of sirtuin 1 and sirtuin 3—the most important and studied sirtuins—at enzyme and protein level in human breast milk. 

In this study, we established methods to measure sirtuin 1 and sirtuin 3 enzyme activities both in the cellular pellet and in the aqueous phase of human breast milk. To the best of our knowledge, there is only one study by Nyárády et al. that examined certain perinatal factors in relation to the amount of sirtuin 3 protein in native breast milk using an ELISA test [22]. The techniques used were adapted from previous studies by our group in human blood (in vivo) [24] and human cultured cells [46] as well as cultured murine neurons in vitro [18]. Sirtuin 3 activity was higher than sirtuin 1 activity, and similar differences were previously found in human blood [24].

Studies by other groups have shown that some parameters in breast milk change with the duration of lactation, for example, the levels of cytokines and growth factor [39], casein and whey protein [47], immunoglobulins [33], and innate immune factors [40]. In some of these studies, the changes were most pronounced in the early phase of lactation. This prompted us to take milk samples for the analysis of sirtuins at different time points of lactation. We chose 7 days (transitional milk), 14 days, 21 days, and 28 days (mature milk), thus covering the complete neonatal period. There was a negative correlation of sirtuin activity and duration of lactation both for sirtuin 1 and sirtuin 3 in the aqueous phase (Figure 1 and Figure 2). No correlation was found for enzyme activities in the pellet and the duration of lactation. Thus, sirtuin activities in the aqueous phase do not simply reflect contents in the cellular pellet, suggesting that sirtuin activity in the aqueous phase does not originate from the cells in breast milk. It is not clear why the protein content of sirtuin 1 and sirtuin 3 in the pellet correlated negatively with the duration of lactation without having an effect on sirtuin activities in the cell pellet (Figure 4b and Figure 6b). Posttranslational modifications of sirtuins may be involved. 

Sirtuin activity in the aqueous phase may originate from the cellular fraction of milk. We therefore tried to correlate sirtuin activities in the aqueous phase with the protein amount in the pellet. No correlation was found (Figure 4 and Figure 6), which may argue against a cellular origin of sirtuins in breast milk. Alternatively, sirtuins in breast milk may be secreted by the mammary gland. Further experimentation is required to solve this issue.

In animal experiments, resveratrol, an activator of sirtuins, fed to mothers and neonatal offspring was able to prevent neurological dysfunction and metabolic syndrome induced by a high-fat diet to mothers and neonates [31,32]. Similarly, the activation of sirtuins may have a beneficial impact on long-term outcome in breast-fed humans.

Mechanistically, sirtuin in breast milk may have a local effect on the gut, regulating for example, metabolic processes and the metabolome, cell signaling, and transport across intestinal membranes. Sirtuins were shown to have an effect on the regulation of proliferation and differentiation of intestinal cells [29], the intestinal microbiota [26,28], and intestinal inflammation [28]. Modulation of the enteric nervous system by sirtuins has been suggested to impact the brain via the microbiota–gut–brain axis [26]. On the other hand, sirtuins in breast milk may be taken up by the intestine and enter the systemic circulation. Sirtuins may then impact cell and organ function and possibly regulate cell programming with long-term effects.

To gain further insight into possible signaling pathways and uptake of sirtuins, animal experimentation is required; alternatively, human intestinal models, for example, organoids, organs on a chip, 3D bio-models [48,49,50], may be used to study the effect and fate of sirtuins in breast milk. High-throughput screening platforms and computer-aided drug design may be helpful tools to mechanistically identify metabolites interacting and modifying sirtuins [51,52].

In future studies, we plan to systematically analyze sirtuins in human breast milk in the context of prematurity, preeclampsia, and HELLP syndrome.

Our study clearly has limitations: the composition of human milk depends on environmental and maternal factors [33,39,53,54], and we tried to control it by careful selection of pregnancies. This restricted the recruitment of appropriate pregnancies/mothers, which resulted in a relatively small number of pregnancies; however, the number of pregnancies was well above the calculated sample size statistically required. 

## 4. Materials and Methods

This study was approved by the ethical board of Hannover Medical School (EC No 8482_BO_K_2019), and written informed consent from all mothers was obtained before participation. 

Inclusion criteria for breast milk collection were full-term or pre-term delivery of a newborn with an estimated hospitalization of more than 28 days to allow best control and immediate processing of breast milk samples. Mothers were mainly non-smoking omnivores. The breast milk was mechanically pumped randomly during the day and stored at −80 °C until it was analyzed. 

Breast milk was subsequently centrifuged at 4 °C, 8875× *g* for 30 min and thus separated into the cell pellet, an aqueous phase, and a lipid layer [45]. Only the aqueous phase and the cell pellet were used in our experiments.

### 4.1. Enzymatic Activity of Sirtuins

The enzyme activities of sirtuin 1 and sirtuin 3 were measured using a modification of the protocol previously used by our group for human blood [24] and human cultured fibroblasts [46], using the Fluorometric Drug Discovery Kit by Enzo Life Sciences (A FLUOR DE LYS ^®^ Fluorescent Assay System, Farmingdale, New York, NY, USA).

For this measurement, the pellet was resuspended in 380 µL of HEPES buffer (110 mM NaCl, 2.6 mM KCl, 1.2 mM KH_2_PO_4_, 1.2 mM MgSO_4_ × 7 H_2_O, 1 mM CaCl_2_, 25 mM HEPES, pH 7.4), and the aqueous phase was directly used. 

After incubation at 37 °C for 15 min, the developer II reagent (76% assay buffer, 20% developer II reagent and 4% nicotinamide) was added to stop the reaction. After further incubation at 37 °C for 45 min, the activity was measured fluorometrically by Tecan Infinite M200 Pro (Männedorf, Switzerland). The same method was used for sirtuin 1 and sirtuin Only the stock solution of the substrate differed between the assays. The measurements were repeated 3 times and replicated at least 3 times.

### 4.2. Protein Expression

Proteins were separated by an 8% sodium dodecyl sulfate polyacrylamide (SDS) page gel to detect sirtuin 1, and a 10% SDS page gel to detect sirtuin The proteins were transferred to a nitrocellulose membrane (GE Healthcare, Chicago, IL, USA) using a semidry blotting method (25 min, 200 mA, Biometra Fastblot, Analytik Jena AG, Jena, Germany). The membrane for sirtuin 1 was blocked for 1.5 h with a blocking solution containing 5% bovine serum albumin (GERBU Biotechnik GmbH, Heidelberg, Germany), and the sirtuin 3 membrane was blocked with pure blocking buffer (LI-Cor, Biosciences, Lincoln, NE, USA). The membranes were incubated with primary antibodies overnight at 4 °C. The protein levels of sirtuin 1 and sirtuin 3 were assessed using primary antibodies at the following dilutions: anti sirtuin 1 mouse 1:1000 (abcam #110304, Cambridge, UK), anti sirtuin 3 rabbit 1:250 (abcam #40963, Cambridge, UK). For internal control (housekeeping protein), anti-vinculin mouse (#V9131 Sigma-Aldrich, St. Louis, MO, USA) was used at the dilution of 1:The secondary antibodies were diluted 1:20,000 for sirtuins and 1:25,000 for the housekeeping protein vinculin (goat anti-mouse, goat anti-rabbit; LI-Cor Biosciences, Lincoln, NE, USA). The membrane with sirtuin 1 was stripped with Thermo Scientific stripping buffer to prepare for measurement of the internal control vinculin as a housekeeper. The photographs were taken by the Li-Cor FC Odyssey^®^ Fc Imaging System (LI-Cor Biosciences, Lincoln, NE, USA) and analyzed with Image Studio Lite 5.2 (LI-Cor Biosciences, Lincoln, NE, USA).

Results were expressed as the ratio between the amount of sirtuin and that of the housekeeping protein vinculin. 

### 4.3. Statistical Analysis 

Based on results in human blood, a sample size of 5 was calculated based on a power of 80% and a level of significance of 5%. No data for sample size calculation in breast milk were available a priori; however, we had experience from previous experimentations in other media/matrices such as blood and cultured cells. 

The statistical analysis was performed by GraphPad Prism 7.2 (GraphPad Software, San Diego, CA, USA). Normal distribution of the values was estimated by the Shapiro–Wilk normality test. Based on the normality test, the mean of enzyme activity was compared among all mothers and the days after delivery using one-way analysis of variance (ANOVA) and *t*-test. A *p*-value < 0.05 was considered statistically significant. 

## 5. Conclusions

In conclusion, we developed analytical procedures to measure sirtuin 1 and sirtuin 3 in the cell pellet and aqueous phase of human breast milk at the enzyme level and in the cell pellet at the protein level. We found a negative correlation between sirtuin activities in the aqueous phase and the duration of lactation in the neonatal period. These assays may form the basis for further studies analyzing the impact of gestational age, mode of delivery, and pregnancy complications on the function of sirtuins. An interesting question is the putative uptake of sirtuins by the systemic circulation of the child, their local effects on the gut function, and their impact on the intestinal microbiome. Further experimentation is required to address these issues.

## Figures and Tables

**Table 1:** Data of the participants.

(**a**)					
	**Mother 1**	**Mother 2**	**Mother 3**	**Mother 4**	**Mother 5**
Age	31	28	37	30	35
Week of gestation	31 + 3	37 + 5	38 + 5	33 + 6	28 + 1
Height in m	1.73	1.60	1.67	1.68	1.76
Weight in kg	75	54	70	75	95
BMI in kg/m^2^	25	21	25	27	31
Pregnancy complications	early rupture of membranes, premature labour	--	polyhydramnion	twin pregnancy, no TTTS	preterm birth
Pre-existing illness	spondylarthritis	--	pelvic vein thrombosis in previous pregnancy	sinus tachycardia, incomplete RBBB	--
Drugs	sulfasalazine	iron	heparin	heparin, iron	--
Smoking in general	no	no	no	no	no
Smoking during pregnancy	no	no	no	no	no
Diet	OV	OV	OV	OV	OV
(**b**)					
	**Mother 6**	**Mother 7**	**Mother 8**	**Mother 9**	**Mother 10**
Age	31	39	33	30	42
Week of gestation	29 + 3	29 + 2	29 + 1	33 + 2	32 + 2
Height in m	1.56	1.89	1.68	1.74	1.71
Weight in kg	67	88	49	82	75
BMI in kg/m^2^	28	25	17	27	26
Pregnancy complications	--	early rupture of membranes	twin pregnancy, hyperemesis gravidarum, cervical insufficiency	--	Vasa praevia
Pre-existing illness	--	--	Intestinal fructose intolerance	hypothyroidism	Hashimoto’s thyroiditis, factor V Leiden deficiency
Drugs	--	unknown	--	L-thyroxin	L-thyroxin
Smoking in general	yes	no	no	no	no
Smoking during pregnancy	yes	no	no	no	no
Diet	OV	OV	OV	OV	OV

Abbreviations: OV (omnivore), TTTS (twin-to-twin transfusion syndrome), RBBB (Right Bundle Branch Block).

## Data Availability

Data supporting the reported results can be found in registered logbooks and are stored on institutional servers.

## References

[1] Weltgesundheitsorganisation, Unicef (2003). Global Strategy for Infant and Young Child Feeding.

[2] Le Doare K., Holder B., Bassett A., Pannaraj P.S. (2018). Mother’s Milk: A Purposeful Contribution to the Development of the Infant Microbiota and Immunity. Front. Immunol..

[3] Hanson L.A., Korotkova M., Lundin S., Håversen L., Silfverdal S., Mattsby-Baltzer I., Strandvik B., Telemo E. (2003). The transfer of immunity from mother to child. Ann. New York Acad. Sci..

[4] Barker D.J. (1995). Fetal origins of coronary heart disease. BMJ.

[5] Beluska-Turkan K., Korczak R., Hartell B., Moskal K., Maukonen J., Alexander D.E., Salem N., Harkness L., Ayad W., Szaro J. (2019). Nutritional Gaps and Supplementation in the First 1000 Days. Nutrients.

[6] Robertson R.C., Manges A.R., Finlay B.B., Prendergast A.J. (2019). The Human Microbiome and Child Growth—First 1000 Days and Beyond. Trends Microbiol. (Regul. Ed.).

[7] Bode L. (2020). Human Milk Oligosaccharides: Structure and Functions. Nestle Nutr. Inst. Workshop Ser..

[8] Seferovic M.D., Mohammad M., Pace R.M., Engevik M., Versalovic J., Bode L., Haymond M., Aagaard K.M. (2020). Maternal diet alters human milk oligosaccharide composition with implications for the milk metagenome. Sci. Rep..

[9] Rinninella E., Raoul P., Cintoni M., Franceschi F., Miggiano G., Gasbarrini A., Mele M.C. (2019). What is the Healthy Gut Microbiota Composition? A Changing Ecosystem across Age, Environment, Diet, and Diseases. Microorganisms.

[10] Samuel T.M., Zhou Q., Munblit D., Thakkar S.K., Verhasselt V., Giuffrida F. (2020). Nutritional and Non-nutritional Composition of Human Milk Is Modulated by Maternal, Infant, and Methodological Factors. Front. Nutr. (Lausanne).

[11] Feldman J.L., Dittenhafer-Reed K.E., Denu J.M. (2012). Sirtuin catalysis and regulation. J. Biol. Chem..

[12] Carafa V., Rotili D., Forgione M., Cuomo F., Serretiello E., Hailu G.S., Jarho E., Lahtela-Kakkonen M., Mai A., Altucci L. (2016). Sirtuin functions and modulation: From chemistry to the clinic. Clin. Epigenet..

[13] Haigis M.C., Sinclair D.A. (2010). Mammalian sirtuins: Biological insights and disease relevance. Annu. Rev. Pathol..

[14] Michan S., Sinclair D. (2007). Sirtuins in mammals: Insights into their biological function. Biochem. J..

[15] Ansari A., Rahman M.S., Saha S.K., Saikot F.K., Deep A., Kim K. (2017). Function of the SIRT3 mitochondrial deacetylase in cellular physiology, cancer, and neurodegenerative disease. Aging Cell.

[16] Chang H.C., Guarente L. (2014). SIRT1 and other sirtuins in metabolism. Trends Endocrinol. Metab..

[17] Jesko H., Wencel P., Strosznajder R.P., Strosznajder J.B. (2017). Sirtuins and Their Roles in Brain Aging and Neurodegenerative Disorders. Neurochem. Res..

[18] Dabke P., Das A.M. (2020). Mechanism of Action of Ketogenic Diet Treatment: Impact of Decanoic Acid and Beta-Hydroxybutyrate on Sirtuins and Energy Metabolism in Hippocampal Murine Neurons. Nutrients.

[19] Buler M., Andersson U., Hakkola J. (2016). Who watches the watchmen? Regulation of the expression and activity of sirtuins. FASEB J..

[20] Merksamer P.I., Liu Y., He W., Hirschey M.D., Chen D., Verdin E. (2013). The sirtuins, oxidative stress and aging: An emerging link. Aging (Albany NY).

[21] Singh A., Kukreti R., Saso L., Kukreti S. (2019). Oxidative Stress: A Key Modulator in Neurodegenerative Diseases. Molecules.

[22] Nyárády K., Turai R., Funke S., Györgyi E., Makai A., Prémusz V., Bódis J., Sulyok E. (2020). Effects of perinatal factors on sirtuin 3, 8-hydroxy-2′- deoxyguanosine, brain-derived neurotrophic factor and serotonin in cord blood and early breast milk: An observational study. Int. Breastfeed. J..

[23] Agarwal A., Gupta S., Sharma R.K. (2005). Role of oxidative stress in female reproduction. Reprod. Biol. Endocrinol..

[24] Potthast A.B., Nebl J., Wasserfurth P., Haufe S., Eigendorf J., Hahn A., Das A. (2020). Impact of Nutrition on Short-Term Exercise-Induced Sirtuin Regulation: Vegans Differ from Omnivores and Lacto-Ovo Vegetarians. Nutrients.

[25] Maissan P., Mooij E.J., Barberis M. (2021). Sirtuins-Mediated System-Level Regulation of Mammalian Tissues at the Interface between Metabolism and Cell Cycle: A Systematic Review. Biology.

[26] Chandramowlishwaran P., Vijay A., Abraham D., Li G., Mwangi S.M., Srinivasan S. (2020). Role of Sirtuins in Modulating Neurodegeneration of the Enteric Nervous System and Central Nervous System. Front. Neurosci..

[27] Ratsika A., Codagnone M.C., O’mahony S., Stanton C., Cryan J.F. (2021). Priming for Life: Early Life Nutrition and the Microbiota-Gut-Brain Axis. Nutrients.

[28] Wellman A.S., Metukuri M.R., Kazgan N., Xu X., Xu Q., Ren N.S.X., Czopik A., Shanahan M.T., Kang A., Chen W. (2017). Intestinal Epithelial Sirtuin 1 Regulates Intestinal Inflammation during Aging in Mice by Altering the Intestinal Microbiota. Gastroenterology (N.Y. 1943).

[29] Li C., Zhou Y., Rychahou P., Weiss H.L., Lee E.Y., Perry C.L., Barrett T.A., Wang Q., Evers B.M. (2020). SIRT2 Contributes to the Regulation of Intestinal Cell Proliferation and Differentiation. Cell. Mol. Gastroenterol. Hepatol..

[30] Zhang K., Zhang X., Lv A., Fan S., Zhang J. (2020). Saccharomyces boulardii modulates necrotizing enterocolitis in neonatal mice by regulating the sirtuin 1/NF-κB pathway and the intestinal microbiota. Mol. Med. Rep..

[31] Sheen J., Yu H., Tain Y., Tsai W., Tiao M., Lin I., Tsai C., Lin Y., Huang L. (2018). Combined maternal and postnatal high-fat diet leads to metabolic syndrome and is effectively reversed by resveratrol: A multiple-organ study. Sci. Rep..

[32] Li S., Yu H., Sheen J., Tiao M., Tain Y., Lin I., Lin Y., Chang K., Tsai C., Huang L. (2017). A maternal high-fat diet during pregnancy and lactation, in addition to a postnatal high-fat diet, leads to metabolic syndrome with spatial learning and memory deficits: Beneficial effects of resveratrol. Oncotarget.

[33] Czosnykowska-Lukacka M., Lis-Kuberka J., Krolak-Olejnik B., Orczyk-Pawilowicz M. (2020). Changes in Human Milk Immunoglobulin Profile During Prolonged Lactation. Front. Pediatr..

[34] Lyons K.E., Ryan C.A., Dempsey E.M., Ross R.P., Stanton C. (2020). Breast Milk, a Source of Beneficial Microbes and Associated Benefits for Infant Health. Nutrients.

[35] Geddes D., Perrella S. (2019). Breastfeeding and Human Lactation. Nutrients.

[36] Pereira C.V., Lebiedzinska M., Wieckowski M.R., Oliveira P.J. (2012). Regulation and protection of mitochondrial physiology by sirtuins. Mitochondrion.

[37] Miller J., Tonkin E., Damarell R.A., McPhee A.J., Suganuma M., Suganuma H., Middleton P.F., Makrides M., Collins C.T. (2018). A Systematic Review and Meta-Analysis of Human Milk Feeding and Morbidity in Very Low Birth Weight Infants. Nutrients.

[38] Twigger A., Hepworth A.R., Lai C.T., Chetwynd E., Stuebe A.M., Blancafort P., Hartmann P.E., Geddes D.T., Kakulas F. (2015). Gene expression in breastmilk cells is associated with maternal and infant characteristics. Sci. Rep..

[39] Munblit D., Treneva M., Peroni D.G., Colicino S., Chow L.Y., Dissanayeke S., Pampura A., Boner A.L., Geddes D.T., Boyle R.J. (2017). Immune Components in Human Milk Are Associated with Early Infant Immunological Health Outcomes: A Prospective Three-Country Analysis. Nutrients.

[40] Trend S., Strunk T., Lloyd M.L., Kok C.H., Metcalfe J., Geddes D.T., Lai C.T., Richmond P., Doherty D.A., Simmer K. (2016). Levels of innate immune factors in preterm and term mothers’ breast milk during the 1st month postpartum. Br. J. Nutr..

[41] George A.D., Gay M.C.L., Trengove R.D., Geddes D.T. (2018). Human Milk Lipidomics: Current Techniques and Methodologies. Nutrients.

[42] Jenness R. (1979). The composition of human milk. Semin. Perinatol..

[43] Melnik B.C., Kakulas F., Geddes D.T., Hartmann P.E., John S.M., Carrera-Bastos P., Cordain L., Schmitz G. (2016). Milk miRNAs: Simple nutrients or systemic functional regulators?. Nutr. Metab..

[44] Bode L., McGuire M., Rodriguez J.M., Geddes D.T., Hassiotou F., Hartmann P.E., McGuire M.K. (2014). It’s alive: Microbes and cells in human milk and their potential benefits to mother and infant. Adv. Nutr. (Bethesda Md.).

[45] Witkowska-Zimny M., Kaminska-El-Hassan E. (2017). Cells of human breast milk. Cell. Mol. Biol. Lett..

[46] Sandvoß M., Potthast A.B., von Versen-Höynck F., Das A.M. (2017). HELLP Syndrome. Reprod. Sci. (Thousand Oaks Calif.).

[47] Gridneva Z., Tie W.J., Rea A., Lai C.T., Ward L.C., Murray K., Hartmann P.E., Geddes D.T. (2018). Human Milk Casein and Whey Protein and Infant Body Composition over the First 12 Months of Lactation. Nutrients.

[48] Madden L.R., Nguyen T.V., Garcia-Mojica S., Presnell S.C., Nguyen D.G., Retting K.N. (2018). Bioprinted 3D Primary Human Intestinal Tissues Model Aspects of Native Physiology and ADME/ Tox Functions. IScience.

[49] Salman M.M., Marsh G., Kusters I., Delince M., Di Caprio G., Upadhyayula S., De Nola G. (2020). Design and validation of a human brain endothelial microvessel-on-a-chip open microfluidic model enabling advanced optical imaging. Front. Bioeng. Biotechnol..

[50] Min S., Kim S., Cho S. (2020). Gastrointestinal tract modeling using organoids engineered with cellular and microbiota niches. Exp. Mol. Med..

[51] Aldewachi H., Al-Zidan R.N., Conner M.T., Salman M.M. (2021). High-Throughput Screening Platforms in the Discovery of Novel Drugs for Neurodegenerative Diseases. Bioengineering.

[52] Salman M.M., Al-Obaidi Z., Kitchen P., Loreto A., Bill R.M., Wade-Martins R. (2021). Advances in Applying Computer-Aided Drug Design for Neurodegenerative Diseases. Int. J. Mol. Sci..

[53] Leghi G.E., Netting M.J., Middleton P.F., Wlodek M.E., Geddes D.T., Muhlhausler A.B.S. (2020). The impact of maternal obesity on human milk macronutrient composition: A systematic review and meta-analysis. Nutrients.

[54] Stinson L.F., Gay M.C.L., Koleva P.T., Eggesbo M., Johnson C.C., Wegienka G., du Toit E., Shimojo N., Munblit D., Campbell D.E. (2020). Human Milk from Atopic Mothers Has Lower Levels of Short Chain Fatty Acids. Front. Immunol..

